# A Meta-Analysis of Anti-Vascular Endothelial Growth Factor Remedy for Macular Edema Secondary to Central Retinal Vein Occlusion

**DOI:** 10.1371/journal.pone.0082454

**Published:** 2013-12-23

**Authors:** Peirong Huang, Wenquan Niu, Zhentian Ni, Renzuo Wang, Xiaodong Sun

**Affiliations:** 1 Department of Ophthalmology, Shanghai First People’s Hospital, School of Medicine, Shanghai Jiao Tong University, Shanghai, China; 2 Eye Research Institute of Shanghai Jiao Tong University, Shanghai, China; 3 State Key Laboratory of Medical Genomics, Ruijin Hospital, School of Medicine, Shanghai Jiao Tong University, Shanghai, China; 4 Department of Hypertension, Ruijin Hospital, School of Medicine, Shanghai Jiao Tong University, Shanghai, China; 5 Department of Surgery, Ruijin Hospital, School of Medicine, Shanghai Jiao Tong University, Shanghai, China; Medical University Graz, Austria

## Abstract

**Background:**

Central retinal vein occlusion (CRVO) associates with severe vision outcome and no proven beneficial treatment. Our meta-analysis intended to appraise the efficacy and safety of anti-vascular endothelial growth factor (anti-VEGF) agents in macular edema (ME) following CRVO.

**Methods:**

Data were collected and analyzed by Review Manager 5.2.1. We employed a random-effects model to eliminate between-study heterogeneity. N_fs_ (called fail-safe number) was calculated to evaluate the publication bias.

**Results:**

We included 5 trials consisting 323 cases and 281 controls. Primary outcomes showed that overall comparison of anti-VEGF agents with placebo control yielded a 374% and 136% increased tendency for a gain of 15 letters or more on Early Treatment Diabetic Retinopathy Study (ETDRS) chart (95% confidence interval [95% CI]: 2.43–9.23; P<0.00001; *I^2^* = 59%, 95% CI: 1.60–3.49; P<0.0001; *I^2^* = 0%, respectively) at 6 and 12 months. Secondary outcomes showed that a 90% and 77% decreased risk at 6 and 12 months for a loss of 15 letters or more. The overall mean difference showed a statistically significance in best-corrected visual acuity (BCVA) on each time point. However, changes of central retinal thickness (CRT) lost significance at 12 months after 6-month as-needed treatment. The incidence of adverse events (AEs) had no statistical difference between anti-VEGF and placebo groups. Subgroup analyses indicated that patients receiving Aflibercept got the highest tendency to gain 15 letters or more (OR = 9.78; 95% CI: 4.43–21.56; P<0.00001). Age controlled analysis suggested a weaken tendency of BCVA improvement in age over 50 (MD = 12.26; 95% CI: 7.55–16.98; P<0.00001). Subgroup analysis by clinical classification showed a strengthen difference of BCVA changes at 6 months in ischemic type (MD = 19.65 letters, 95% CI: 13.15 to 26.14 letters, P<0.00001).

**Conclusions:**

Our results showed that anti-VEGF agents were superior to placebo in CRVO-ME treatment with no statistically significant AEs, especially in younger people and for ischemic type.

## Introduction

Retinal vein occlusion (RVO) has been the second most frequent cause of retinal vascular disease [1], [2]. Although central retinal vein occlusion (CRVO) occurs comparatively less than branch retinal vein occlusion (BRVO), it associates with severe vision outcome and has no proven beneficial treatment by far. The exact pathogenesis of CRVO remains incompletely understood while some underlying etiological factors were implicated (Table S1) [3]–[5]. Macular edema (ME) following CRVO leads to a consequent reduction of visual acuity, especially the ischemic subtype. The recommended treatment of CRVO became observation instead of grid laser after the NEI-sponsored multicenter randomized controlled trials (RCT) Central Vein Occlusion Study in 1994 concluded the inefficiency of grid photocoagulation in either preserving or improving visual acuity in CRVO-ME eyes [6]. Ever since then various medical and surgical treatments have been attempted and pharmacologic agents including anti-vascular endothelial growth factor (anti-VEGF) and steroids demonstrated great promise, showing improved visual acuity and ME regression [7]–[9].

CRVO patients present higher VEGF concentration in the ocular fluids, mediating active intraocular neovascularization and permeability [10]. Anti-VEGF injection inhibits VEGF-driven neovascularization in vitro as well as in vivo [11], [12]. This beneficial therapeutic choice leads to several anti-VEGF agents such as Pegaptanib (Macugen®; Eyetech Pharmaceuticals, New York, NY), Bevacizumab (Avastin®; Genentech, Inc., South San Francisco, CA), Ranibizumab (Lucentis®; Novartis Pharmaceuticals, East Hanover, NJ) and Aflibercept (EYLEATM®, also named VEGF Trap-eye; Regeneron Pharmaceuticals Inc., Tarrytown, New York, USA) using off-lab [13]. The main stream of anti-VEGF agents involved in our study is listed in Table 1. Here, we aim to conduct a meta-analysis to gain better perspective of the efficacy and safety of anti-VEGF agents for CRVO-ME. The Cochrane collaboration conducted a review on this topic in 2010 but there was no meta-analysis performed in short of enough RCT meeting the inclusion criteria [14]. As far as we know, we are the first to quantify the effect of anti-VEGF functionally and anatomically, providing ophthalmologists with stronger clinical evidence. Meanwhile, adverse events were also studied in this report.

**Table 1:** Anti-vascular endothelial growth factor included in this meta-analysis.

Agents	Class	Initial U.S. approval time	FDA approval use
**Pegaptanib sodium (Macugen®)**	Aptamer	2004	Neovascular (Wet) Age-Related Macular Degeneration (AMD)
**Ranibizumab (Lucentis®)**	VEGF-specific antibodies	2006	Neovascular (Wet) Age-Related Macular Degeneration (AMD)
			Macular Edema Following Retinal Vein Occlusion (RVO)
			Diabetic Macular Edema (DME)
**Bevacizumab (Avastin®)**	VEGF-specific antibodies	2004	Non-Squamous Non-Small Cell Lung Cancer (NSCLC)
			Metastatic Colorectal Cancer (mCRC)
			Metastatic Renal Cell Carcinoma (mRCC)
			Glioblastoma
**Aflibercept (EYLEATM®), also known as VEGF Trap-Eye**	ImmunoglobulinG-VEGF receptor fusion protein	2011	Neovascular (Wet) Age-Related Macular Degeneration (AMD)
			Macular Edema Following Central Retinal Vein Occlusion (CRVO)

## Methods

This meta-analysis abides by the statement of the Preferred Reporting Items for Systematic Reviews and Meta-analyses (PRISMA) [15].

### Literature Search

Two investigators (PR. Huang and ZT. Ni) participated in the literature search via PubMed (http://www.ncbi.nlm.nih.gov/pubmed/), Embase (http://www.embase. com), The RCCTs in Cochrane Central Register of Controlled Trials, the metaRegister of Controlled Trials (www.controlled-trials.com), and ClinicalTrials.gov (www.clinicaltrials.gov) till January 2013. The search term used were “central retinal vein occlusion”, “macular edema”, “anti-VEGF”, “pegaptanib”, “Macugen”, “bevacizumab”, “Avastin”, “ranibizumab”, “Lucentis”, “aflibercept”, “Trap-eye” and “clinical trial” in various combinations. Related citations in Pubmed and references of related studies were also incorporated. Searches were limited in articles written in English literature and done in human species. Final selection was made after the two investigators reached an agreement. If two or more studies based on the same population, the more comprehensive one was included.

### Inclusion and Exclusion Criteria

Studies were included if they satisfied the criteria below: (1) randomized controlled clinical trial; (2) comparing anti-VEGF with placebo treatment for CRVO-ME; (3) proportion of gain or lose more than 15 letters, changes of BCVA and CRT of treatment group and placebo controls available for calculating odds ratio (OR), mean difference (MD) and 95% confidence interval (95% CI). Studies were excluded if they were retrospective, non-controlled, nonrandomized, in non-English languages or abstracts from meetings.

### Study Selection

Ten potential RCCT trials were identified. One of them compared two different doses of intraviteal Ranibizumab, one of them compared intraviteal Bevacizumab (IVB) to intraviteal triamcinolone acetonide (IVT), and one of them compared two patterns of PRN (Pro Re Nata) injections (monthly or quarterly intervals). They were all excluded according to the inclusion criteria. The 6-month results of two studies were excluded and only the final results were included to avoid repetition. At last, five studies were identified after investigators’ discussion.

### Data Extraction

WQ. Niu and PR. Huang separately collected the following data from all included researches: (1) proportion of a gain or loss of 15 letters or more from baseline; (2) means and standard deviations (SDs) of changes from baseline in best-corrected visual acuity (BCVA) in Early Treatment Diabetic Retinopathy Study (ETDRS) letters equivalents; (3) means and standard deviations (SDs) of changes from baseline in central retinal thickness (CRT) in µm; (4) characteristics of the included studies, eg, name or first author of the study, year and country, main inclusion and exclusion criteria, different treatment methods, number of eyes, mean age of patients, sex ratio, follow-up points, etc. Inadequate data were obtained from trial authors.

### Outcome Measurement

The primary outcome was the proportion of cases and controls with an increase from baseline in BCVA of larger than or equal to 15 letters on the ETDRS chart at four meters after 6 and 12 months of follow-up periods. Gaining 15 letters has been the standard primary outcome measure for evaluating the efficacy of treatments in retinal disorders [16]. The secondary outcomes included: (1) the proportion of patients losing 15 letters or more ETDRS letters compared to baseline at 6 and 12 months; (2) mean changes of visual acuity from baseline with different inventions, indicating functional improvement; (3) mean changes of central retinal thickness (CRT) from baseline with different inventions on ocular coherence tomography (OCT), indicating anatomical improvement. We chose 1,6 and 12 months as the time point for analyze as they both satisfied common presence among the studies and gave a representative understanding of short-term, mid-term and long-term efficacy of the intervention. In Wroblewski et al. and Epstein et al. studies, the 6-week results substituted the results of 1-month. We searched for any ocular and non-ocular adverse events (AEs) specifically aiming to glaucoma, cataract, endophthalmitis and Antiplatelet Trialists’ Collaboration arterial thromboembolic events (APTC ATEs). Then we compared difference in frequencies of the most frequent ones between the study and placebo group.

### Quality Assessment

There are various kinds of tools to assess studies, of which the Jadad score is frequently used for RCTs [17]. In our meta-analysis, the methodological efficiency of studies were analyzed for their qualities based on the modi fied Jadad scoring system developed by Crowther et al [18]. Efficiency assessment was also performed separately by two investigators and the results were consistent. The details are described in Table 2.

**Table 2:** Quality assessment of included RCTs in this meta-analysis.

Author (year)	Question 1	Question 2	Question 3	Question 4	Question 5	Question 6	Question 7	Score
**Wroblewski et al. (2009)**	Yes	Yes	No	Yes	Yes	No	Yes	5
**Cruise study (2011)**	Yes	Yes	No	Yes	Yes	No	Yes	5
**Epstein et al. (2012)**	Yes	Yes	No	Yes	Yes	No	Yes	5
**Copernicus study (2013)**	Yes	Yes	No	Yes	Yes	No	Yes	5
**ROCC study (2010)**	Yes	No	No	Yes	Yes	No	Yes	4

The modified Jadad scoring system for randomized controlled trials (Crowther M et al. Blood. 2010; 116∶3140–3146).

**Question 1.** Was the study described as randomized? If yes, score 1 point.

**Question 2.** If yes to question 1, was an appropriate randomization sequence described and used (eg, table of random numbers, computer generated, etc.)? If yes, score 1 point.

**Question 3.** If yes to question 1, was an inappropriate method to generate the sequence of randomization used (patients were allocated alternately, or according to date of birth, hospital number, etc.)? If yes, subtract 1 point.

**Question 4.** Was the study described as double blinded? If yes, score 1 point.

**Question 5.** If yes to question 4, was an appropriate method of blinding used (eg, identical placebo, active placebo, dummy, etc.)? If yes, score 1 point.

**Question 6.** If yes to question 4, was an inappropriate method for blinding used (eg, comparison of tablet vs. injection with no double dummy)? If yes, subtract 1 point.

**Question 7.** Were the withdrawals and dropouts described? If yes, score 1 point.

### Statistical Analysis

Data were collected and analyzed by Review Manager 5.2.1. Mantel-Haenszel was used for dichotomous variables as odds ratios (ORs) with 95% confidence intervals (CIs) and inverse variance was used for normally distributed continuous variables. The difference between anti-VEGF treatment modality and placebo control was displayed by forest plot. *I^2^* statistic (ranging from 0 to 100%) was used to quantify between-study heterogeneity rather than chance (*I^2^* = 0–25%, no heterogeneity; *I^2^* = 25–50%, moderate heterogeneity; *I^2^* = 50–75%, large heterogeneity; *I^2^* = 75–100%, extreme heterogeneity) [19].

We employed N_fs_ (called fail-safe number) to evaluate the publication bias. If the value of N_fs_ for one comparison turned out to be smaller than the number of included trials, it implied a significant publication bias. We calculated the N_fs_ significance by the formula N_fs_0.05 =  (∑Z/1.64)^2^-k, where k equals to the number of observed studies [20]–[23].

## Results

### Study Characteristics

**Figure 1:**
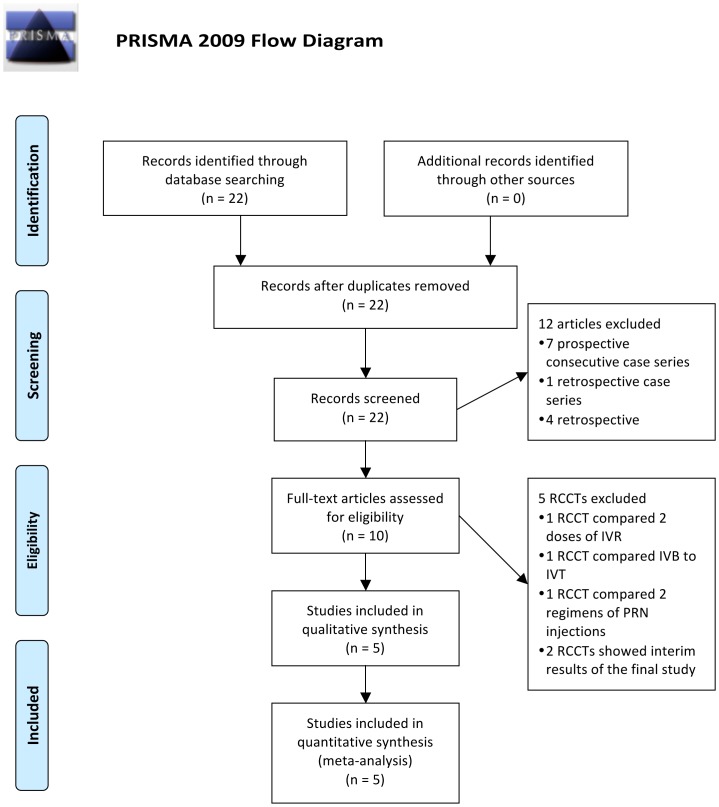
Flow chart of search strategy and study selection. RCCT: Randomize Case Controlled Trial, IVR: IntraViteal Ranibizumab, IVB: IntraViteal Bevacizumab, IVT: IntraViteal Triamcinolone acetonide (IVT), PRN: Pro Re Nata.

A flow chart schematizing the filteration process is presented in Figure1. There were five studies [24]–[28] with a total 604 CRVO-ME eyes (treatment group of 323 patients and 281 controls) involved in this meta. Each trial went through methodological quality assessment, and got a jaded score as described (Table 2). All five RCTs proved to be of high qualification. Despite the ROCC study got 4 points, the others all got full scores. Detailed information is described in Table3.

**Table 3:** The baseline characteristics of all qualified studies in this meta-analysis.

Study	Year and place	Major Inclusion criteria	Major exclusion criteria	Number of eyes	Intervention groups	Mean age (yrs)	Percentage of males	Follow up visits
**Wroblewski et al.** [**24**]	2009, USA	Aged ≥50 yrs	Eyes with a brisk afferent pupillary defect, vitreous hemorrhage	Group 1. n = 33	Group1.Pegaptanib sodium(0.3 mg)-baseline, wks6,12,18,24	64	45.45	Wks1,3,6,12,18,24,30
		Duration ≤6 mos	Evidence of any neovascularization involving the iris, disc, or retina	Group 2. n = 33	Group2. Pegaptanib sodium(1 mg)-baseline, wks6,12,18,24	64	54.54	
		BCVA 65-20 letters, fellow eye ≥35 letters	Signs of old RVO in the study eye, or diabetic retinopathy	Group 3. n = 32	Group3.Placebo	59	59.38	
		CRT ≥250 µm	Subtenon corticosteroid administration	Total: 98				
			Prior panretinal or sector scatter photocoagulation					
			Any other clinically significant concomitant ocular diseases					
**ROCC study ** [**25**]	2010,Norway	Previously untreated CRVO-ME	Any concomitant ocular disease	Group 1. n = 16	Group 1. Placebo	72(52–88)	55.2	Mos1,2,3,4,5,6
		Symptom duration ≤6 mos	Uncontrolled glaucoma, filtration surgery, corneal transplantation	Group 2. n = 16	Group 2. Ranibizumab(0.5 mg)-baseline, mos1,2			
		Age ≥50 yrs	Prior treatment of macular disease	Total: 32				
		BCVA between ≤73 and ≥6 letters	Cataract surgery 3 mos prior to baseline or aphakia					
			Cataract or diabetic retinopathy in rapid progression					
			Vitreous hemorrhage or previous RRD					
			Pregnant					
			Receive treatment for active systemic infection					
			Medication known to be toxic to the eye					
			Contraindication for the use of an investigational drug					
			Hypersensitivity or allergy to fluorescein					
**Cruise study ** [**26**]	2011,USA	≥18 yrs	Prior episode of RVO	Group 1. n = 130	Group 1. Placebo	65.4±13.1	55.4	Day7 and mos1–12
		Diagnosed within 12 mos before study initiation	Panretinal scatter photocoagulation or sector laser photocoagulation within 3 mos	Group 2. n = 132	Group 2. Ranibizumab(0.3 mg)-baseline, mos1,2,3,4,5	69.7±11.6	53.8	
		BCVA 20/40-20/320	>10-letter improvement in BCVA between screening and day0	Group 3. n = 130	Group 3. Ranibizumab(0.5 mg)-baseline, mos1,2,3,4,5	67.6±2.4	61.5	
		Mean central subfield thickness ≥250 µm	History of radial optic neurotomy or sheathotomy	Total: 392	Mos6–11			
			Prior anti-VEGF treatment in study or fellow eye within 3 mos, systemic anti-VEGF or pro-VEGF treatment within 6 mos		Monthly intraocular ranibizumab if study eye BCVA≤20/40 or CRT≥250 µm			
			Intraocular corticosteroid use					
			Brisk afferent pupillary defect					
			Laser photocoagulation for ME within 4 mos					
			Evidence of any diabetic retinopathy					
			CVA or MI within 3 mos					
			History or presence of wet or dry AMD					
**Epstein et al.** [**27**]	2012, Sweden	Duration ≤6 mos	CRVO with neovascularisation	Group 1. n = 30	Group 1. Placebo	70.4±10.4	56.7	Wks6,12,18,24
		BCVA between 15–65 letters	Any previous treatment for CRVO	Group 2. n = 30	Group 2. Bevacizumab(1.25 mg)-baseline, mos1,2	70.6±12.6	63.3	
		Mean central subfield thickness	Glaucoma with advanced visual field defect or uncontrolled ocular hypertension>25 mmHg despite full therapy	Total: 60		70.5±12.6	71.6	
		≥300 µm by OCT	Vascular retinopathy of other causes					
			Intraocular surgery during the previous 3 mos					
			Myocardial infarction or stroke during the last 12 mos					
**Copernicus study ** [**28**]	2013, California	Central subfield retinal thickness ≥250 µm by OCT	History or presence of age-related macular degeneration (AMD, dry or wet form) that significantly affected central vision	Group 1. n = 73	Group 1. Placebo	67.5(14.3)	52.0	Mos1–13
		Center-involved CRVO-ME	Diabetic ME or diabetic retinopathy and infectious blepharitis, keratitis, scleritis, or conjunctivitis	Group 2. n = 114	Group 2. Aflibercept(2 mg)-baseline, mos1,2,3,4,5	65.5(13.6)	61.0	
		Diagnosed within 9 mos	Any ocular disorders that could confound interpretation of study results	Total: 189	Mos6–13	66.3(13.9)	57.0	
		Aged ≥18 yrs	Previous use of intraocular corticosteroids or use of periocular corticosteroids within the 3 mos		PNR: 1 aflibercept(2 mg) injection if ≥50 µm increase in CRT, persistent edema ≥250 µm, decrease of BCVA≥5 letters			
		BCVA of 20/40-20/320 in the study eye	Iris neovascularization, vitreous hemorrhage, traction retinal detachment, or preretinal fibrosis involving the macula		Placebo injection if retreatment not indicated			
			Any previous treatment with antiangiogenic drugs					
			Prior panretinal or macular laser photocoagulation					

BCVA: best-corrected visual acuity, CRT: central retinal thickness, RVO: retinal vein occlusion, CRVO-ME: macular edema following central retinal vein occlusion, RRD: rhegmatogenous retinal detachment, AMD: age-related macular degeneration, MI: myocardial infarction, CVA: cerebrovascular accident, letter: ETDRS (Early Treatment Diabetic Retinopathy Study) letter score, wks: weeks, mos: months, yrs: years.

### Intervention Arms

All of the included trials had consistent treatment arms comparing monthly anti-VEGF agents to placebo injections in the first three months. During month 3 to 6, ROCC study and Epstein study stops injection while Wrobleski et al, Cruise and Copernicus study continued to have four consistent monthly injections until the as-needed treatment period during six to twelve or thirteen months.

### Intervention Results

The results of different follow-up points were reported respectively. Four trials (except ROCC study) all reported the proportion of patients gaining or losing 15 letters or more at 6 months. All studies reported the changes of BCVA in ETDRS and CRT at 6 months. Only Cruise and Copernicus study reported outcomes in details at 12 months [26], [28].

### Pooled Analyses - 15 Letters or More Gain in Visual Acuity (ETDRS Chart)

**Figure 2:**
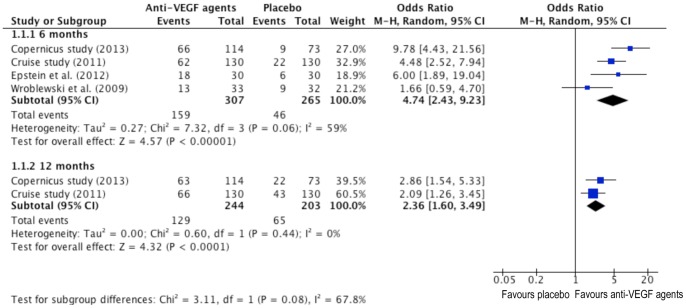
Forrest plots for the proportion of patients with an improvement from baseline in best-corrected visual acuity (BCVA) of greater than or equal to 15 letters on Early Treatment in Diabetic Retinopathy Study (ETDRS) Chart at six and twelve months between anti-VEGF and placebo group.

The difference in proportion of patients gaining 15 letters or more with its odds ratio (OR) and 95% confidence interval (CI) bounds is pointed out in the forest plot (Figure 2). The dots and the whiskers represent the OR and the associated 95% CI respectively. Values to the right of the longitudinal line at 1 show larger proportion of people gaining 15 letters or more in the treatment groups, and values to the left of the line show larger proportion in placebo groups. The subtotal rows stand for the overall values for individual follow-up points. Confidence interval bounds without cutting the line at 1 imply that results are statistically significant at the level of 0.05.

At 6 months, the comparison of anti-VEGF events with placebo injections in 15 letters or more gain in visual acuity generated a significant 374% increased tendencies (P<0.00001) (Figure 2.1.1.1). The *I^2^* value showed large between-study heterogeneity (*I^2^* = 59%, P = 0.06). Only two studies expressed results at 12 months. The comparison of anti-VEGF events with placebo injections in 15 letters or more gain in visual acuity generated a significant 136% increased risk (P<0.0001) (Figure 2.1.1.2). The *I^2^* value showed no between-study heterogeneity (*I^2^* = 0%, P = 0.44).

### Pooled Analyses - 15 Letters or More Lose in Visual Acuity (ETDRS Chart)

**Figure 3:**
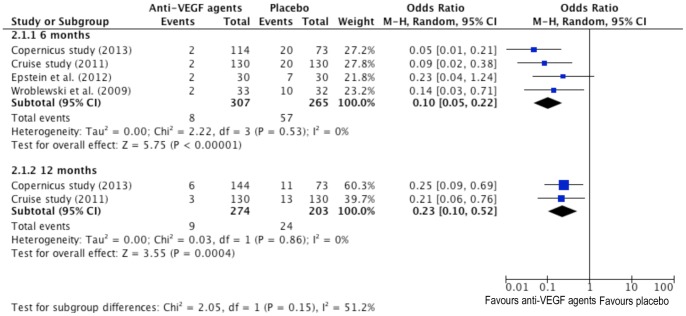
Forrest plots for the proportion of patients with a loss from baseline in best-corrected visual acuity (BCVA) of greater than or equal to 15 letters on Early Treatment in Diabetic Retinopathy Study (ETDRS) Chart at six and twelve months between anti-VEGF and placebo group.

Figure 3 demonstrates the forest plot of proportion of patients losing 15 letters or more results comparing anti-VEGF to placebo controls.

At 6 months, the comparison of anti-VEGF events with placebo injections in 15 letters or more lose in visual acuity resulted a significance of 90% lowered risk (P<0.00001) (Figure 3.2.1.1). The value of *I^2^* informed no between-study heterogeneity (*I^2^* = 0, P = 0.53). Only two studies expressed results at 12 months. The comparison of anti-VEGF events with placebo injections in 15 letters or more lose in visual acuity generated a significant 77% lower risk (P = 0.0004) (Figure 3.2.1.2). The *I^2^* statistic also indicated no between-study heterogeneity (*I^2^* = 0%, P = 0.86).

### Pooled Analyses - Best-Corrected Visual Acuity

**Figure 4:**
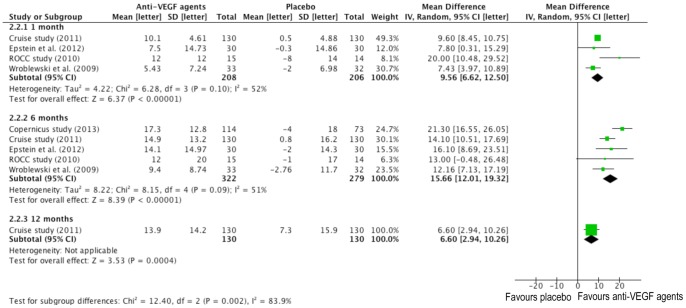
Forrest plots for the mean visual acuity change on Early Treatment in Diabetic Retinopathy Study (ETDRS) Chart at one, six and twelve months between anti-VEGF and placebo group.

The difference in BCVA (on ETDRS chart) with its mean difference (MD) and 95% confidence interval (CI) bounds is pointed out in the forest plot (Figure 4). The dots and the whiskers represent the MD and the associated 95% CIs respectively. Values to the right of the longitudinal line at 0 stand for bigger changes in BCVA in the treatment group and values to the left of the line show bigger changes in the placebo groups.

At one month, all studies except Copernicus study (data not available) showed improvement in BCVA in the anti-VEGF agents group and the summary mean difference (9.56 ETDRS letters) was statistically significant (95% CI: 6.62–12.51; P<0.00001) with large heterogeneity (*I^2^* = 52%, P = 0.10) (Figure 4.2.2.1); At six months, the combined mean difference in BCVA was more statistically significant (15.66 ETDRS letters, 95% CI: 12.01 to 19.32 ETDRS letters) in favor of anti-VEGF treatment with large heterogeneity (*I^2^* = 51%, P = 0.09) (Figure 4.2.2.2). Only Cruise study provided full results at 12 months (Figure 4.2.2.3). The mean difference in BCVA (6.60 ETDRS letters) was statistically significant (95% CI: 2.94 to 10.26 ETDRS letters; P = 0.0004) for the anti-VEGF injection group compared to the control group, which was smallest of the three time points.

### Pooled Analyses - Central Retinal Thickness

**Figure 5:**
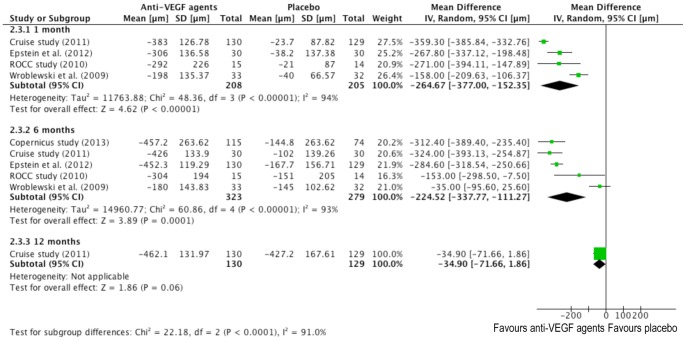
Forrest plots for the mean change in central retinal thickness (µm) at one, six and twelve months between anti-VEGF and placebo group.

The forest plot of CRT results in contrast of the two therapeutical groups is demonstrated in Figure 5. It can be interpreted in a similar way of Figure 4 except the results are in µm. Values to the left of the longitudinal line at 0 show greater ME regression in the treatment group, and values to the right of the line show larger changes in placebo groups.

At one month, nearly all studies (data not available in Copernicus study) demonstrated great regression in CRT in the treatment group. The combined MD in CRT (−264.67 µm) was statically significant (95% CI: −377 to −152.35 µm) in favor of anti-VEGF agents with extreme heterogeneity (*I^2^* = 94%, P<0.00001). At 6 months, the summary mean difference for all studies consistently showed a favorable response to anti-VEGF agents treatment (−224.52 µm) which was statically significant (95% CI: −337.77 to −111.27 µm), also with extreme heterogeneity *I^2^* = 93%). Only Cruise study provided full comparative data at 12 months, with a summary mean difference in CRT of −34.90 µm, which was statistically significant (95% CI: −71.66 to 1.86 µm).

### Adverse Effects

There was no sufficient data about adverse effects, restricting the ability of a meta-analysis to evaluate the efficacy of adverse effects occurring at different follow-up points. Neovascularization (including iris neovascularization, retinal neovascularization, vitreous haemorrhage and tractional retinal detachment) was the most frequently observed adverse effect, which had a higher tendency in the placebo group (mean, 11.71%; SD, 4.88%) than in the treatment group (mean, 5.14%; SD, 4.88%; P = 0.1489). So was glaucoma (mean, 1.13%; SD, 1.77% vs. mean, 0.16%; SD, 0.36%; P = 0.284). On the other hand, endophthalmitis, cataract and retinal artery or vein thrombosis were more observed in treatment group, but all of which were not statically significant (P = 0.3739, 0.4232, 0.3415, respectively). They could be related to improper procedure or to drugs. There were rare non-ocular serious adverse events potentially related to anti-VEGF agents. The Antiplatelet Trialists’ Collaboration Arterial Thromboembolic Events (APTC ATEs) including myocardial infarction, ischemic stroke, vascular deaths were similar in both groups (P = 0.914).

### Subgroup Analyses

Numerous factors including different kind of anti-VEGF types, age of inclusion creteria, clinical subtype (ischemic and non-ischemic) might bias the summary combination, thus we did separate analyses in these aspects.

In view of anti-VEGF agents at month 6, Aflibercept got the highest tendency to gain 15 letters or more in visual acuity after treatment (OR = 9.78; 95% CI: 4.43–21.56; P<0.00001) followed by Bevacizumab (OR = 6.00; 95% CI: 1.89–19.04; p = 0.002). Ranibizumab had a moderate tendency (OR = 4.48; 95% CI: 2.52–7.94; P<0.00001) and Pegaptanib showed least tendency (OR = 1.66; 95% CI: 0.59–4.7; p = 0.34). On the other hand, Aflibercept got the lowest tendency to lose 15 letters or more in visual acuity after treatment (OR = 0.05; 95%CI: 0.01–0.21; P<0.0001) followed by Ranibizumab (OR = 0.09; 95%CI: 0.02–0.38; p = 0.001) and Pegaptanib (OR = 0.14; 95%CI: 0.03–0.71; p = 0.02). Bevacizumab seemed to have no statistically significant effect on reliving visual acuity lost (OR = 0.23; 95% CI: 0.04–1.24; p = 0.09). On month 12, Aflibercept still did better than ranibizumab in 15-letter gain (OR = 2.86; 95% CI: 1.54–5.33; p = 0.0009 vs. OR = 2.09; 95% CI: 1.26–3.45; p = 0.004, respectively) and 15-letter lose (OR = 0.25; 95% CI: 0.09–0.69; p = 0.008 vs. OR = 0.21; 95% CI: 0.06–0.76; p = 0.02, respectively).

At the time of onset, 90% of patients are over 50 years [29]. When it occurs to younger patients, an associated inflammatory or coagulopathy cause should be considered [30], [31]. Since the prevalence of CRVO increases by age and the disease results from different causes, we choose age of fifty as the division boundary in placebo controls to separate the trials into two subgroups to avoid the miss-classification. At month six, the tendency of mean changes of BCVA in ETDRS letters was weakened in age>50 subgroups (MD = 12.26; 95% CI: 7.55–16.98; P<0.00001).

The gender ratio of CRVO in male and female is about 1∶1 [32], while all the studies included in our meta-analyze are >50%, thus we made no subgroup analyze.

The natural history of ischemic and non-ischemic subtype of CRVO differs [33]. Ischemic type is associated with a poor visual prognosis while non-ischemic type has a better one. The ratio of ischemic and non-ischemic is about 1∶4. We chose 20% as the division boundary to separate the trials into two subgroups. A strengthen difference of BCVA changes at 6 months in ischemic subgroup (MD = 19.65 ETDRS letters, 95% CI: 13.15 to 26.14 ETDRS letters, P<0.00001) could be observed. So was the CRT (MD = −245.14; 95% CI: −399.44 to −90.85 µm) at 6 months.

The fail-safe number (N_fs_) was calculated and demonstrated in Table 4. All the N_fs_ values were bigger than the number of observed studies included in the meta-analyses, which implied a non-significant publication bias.

**Table 4:** False-safe number.

Comparison items	N_fs_
**15 or more gain at months 6**	77.11
**15 or more gain at months 12**	12.25
**15 or more lose at months 6**	43.90
**15 or more lose at months 12**	7.37
**BCVA at month 1**	260.46
**BCVA at month 6**	273.54
**CRT at month 1**	728.31
**CRT at month 6**	497.67

N_fs_: fail-safe number, BCVA: best-corrected visual acuity, CRT: central retinal thickness.

## Discussion

We are the first to conduct a meta-analysis of anti-VEGF agents’ applications in the treatment fields of CRVO-ME. In short, our study suggests that the anti-VEGF agents appeared to be of high efficacy and low adverse events in both short and long term.

CRVO treatment is more focused on the complications than on the disease itself [34]. ME secondary to CRVO is found to be a crucial factor of sudden vision loss clinically. Extensive research has been undergoing for tens of years to understand the exact pathogenesis and potential treatment regimens to stabilize and prevent CRVO-ME.

Macular grid photocoagulation had been universally accepted as the therapeutically standard regimens of CRVO-ME until the Central Vein Occlusion Study Group conducted a RCT in 1995 showing that grid laser did not demonstrate better visual acuity results [35]. Since then, observation has been the standard management of CRVO-ME. Recent progress has drastically shifted the treatment regiments from laser to drugs. Intravitreal triamcinolone acetonide was proved to be effective by the SCORE study [9] but was generally associated with well-known adverse events, eg, cataract and glaucoma. Promising results of case series and randomized trials have been found in treating ME following CRVO by intravitreal anti-VEGF drugs both functionally and structurally.

The genuine motive of this study was to explore the superiority of anti-VEGF drugs to placebo controls. Despite the different kind of anti-VEGF agents, they all showed significant consistency in gaining visual acuity and reducing CRT at 1 and 6 months compared to placebo groups. At 6 months, monthly anti-VEGF injections showed substantial functional improvement with mean difference of 15.66 letters (approximately 3 lines, P<0.00001), which was also in accordance with the primary outcome. In Cruise and Copernicus study, both groups had dramatic reduction in ME after 6-month anti-VEGF treatment as needed without significant difference at 1 year. However, unlike the mean CRT, the proportion of patients receiving 15 letters or more from baseline was significantly higher in treatment group (P<0.001). This phenomenon suggested that monthly treatment for 6 months+PRN (Pro Re Nata) was better than observation for 6 months+PRN (Pro Re Nata). Vision loss was irreversible unlike CRT.

In the treatment of CRVO-ME, It had long been appreciated that there was an inverse relationship between OCT-measured retinal thickness and visual acuity until the SCORE Study clarified a statistically significant but modest correlation between them at the baseline (r = −0.27, coefficient of determination R^2^<10%) [36]. Or, more specifically, macular thickness acted more like one of several variables in a complex that affected visual acuity in an incompletely understood relationship. Other variables included age and duration of ME. There’s no report assessing the relationship of CRT change to VA change after treatment in CRVO-ME. Similar study in diabetic macular edema following focal laser treatment demonstrated that the correlation coefficients between them were also modest at each follow-up point [37]. Other reported variables affecting the VA outcomes included the integrity of the outer photoreceptors [38]. In short, OCT-measured CRT can be used as a useful tool to detect and monitor the severity of macular edema rather than a reliable surrogate for visual acuity measurements in clinic at the baseline or during the follow-up periods.

The rising popularity of anti-VEGF drugs came along with concerns about its safety in clinical use. Previous numerous studies of VEGF inhibitions applied in DME showed low incidence of serious side effects like infection and elevated intraocular pressure. The application in CRVO-ME showed the same tendency. The main side effects concluded in our study showed that both cataract and endophthalmitis incidence were very low which could have been due to procedure (intraocular injections) which were not significant. These complications can be largely avoided through standard sterilization and more practice. Compared to placebo group, anti-VEGF group had lower tendencies in neovascularization (P = 0.1489) and glaucoma (P = 0.284). Meanwhile, there were few nonocular adverse events potentially related to anti-VEGF agents including the APTC ATEs. All the including studies showed that anti-VEGF treatment appeared to be safe and generally accepted in 12 months follow-up period.

In brief, our study suggested that all anti-VEGF agents identified to be a more efficacious therapy for CRVO-ME than placebo. Aflibercept seemed to be most effective in improving visual acuity but needed more trials to prove. However, the benefit of anti-VEGF drugs over 1 year was not reported by current studies.

The meta-analysis should be interpreted considering its inherent limitations. The true shortcoming of this study is the small sample size of four different anti-VEGF agents from different company. Secondly, the effectiveness and safety over longer periods of follow-up have yet to be determined since only two studies reported outcomes on 1 year. Thirdly, diabetic retinopathy was excluded in some studies whereas it was one of the established risk factors for CRVO [39].

Despite limitations mentioned afore, the result of this meta-analysis is useful in clinic, providing precious and preliminary data for therapeutical choice. Our study suggests that anti-VEGF agents yield better visual outcomes and achieve more significant edema regression compared to placebo in the first 6 month. Anti-VEGF treatment as needed is necessary to maintain the stable outcome. Early aggressive treatment is helpful for visual acuity improvement. However, many questions remain unsolved such as the regimens after 1 year. Thus, further studies are anticipated to evaluate a longer-term effect of anti-VEGF agents in CRVO-ME.

## Supporting Information

Table S1 **Factors implicated in the pathogenesis of central retinal vein occlusion.**
(DOCX)

Checklist S1 **PRISMA Checklist**
(DOC)
