# Supplementary material for: A Meta-Analysis of Anti-Vascular Endothelial Growth Factor Remedy for Macular Edema Secondary to Central Retinal Vein Occlusion
Source: PLoS One. 2013 Dec 23;8(12):e82454. doi: 10.1371/journal.pone.0082454 (PMC3871640; doi:10.1371/journal.pone.0082454)
Supplement: Table S1 — Factors implicated in the pathogenesis of central retinal vein occlusion. (DOCX) [file pone.0082454.s001.docx]

**Table S1.** Factors implicated in the pathogenesis of central retinal vein occlusion.

| 1. Venous thrombosis (in the region of and just posterior to the lamina cribrosa) |
| --- |
| 2. Arteriovenous crossing compression |
| 3. Chronic open-angle glaucoma |
| 4. Inflammation |
| 5. Systemic factors   - Age - Hypertension - Diabetes - Hyperlipidaemia - Hyperviscosity cardiovascular disease |
| 6. Hereditary alterations in the coagulation pathways   - Activated protein C resistance (factor V Leiden mutation) - Deficiencies of anticoagulant proteins (anti-thrombin III, protein C, protein S, etc.) |
